# Atzumi for migraine: a new era of needle-free dihydroergotamine delivery

**DOI:** 10.1097/MS9.0000000000003652

**Published:** 2025-07-28

**Authors:** Areeba Aamir Ali Basaria, Santosh Sah, Hansa Ratnani, Ismat Fatima, Hermann Yokolo

**Affiliations:** a Department of Medicine, Dow University of Health Sciences, Karachi, Pakistan; b Department of Medicine, Jinnah Sindh Medical University, Karachi, Pakistan; c Department of Research, Medical Research Circle (MedReC), Goma, DR Congo

**Keywords:** acute treatment, atzumi, dihydroergotamine, drug delivery, intranasal therapy, migraine

## Abstract

Migraine continues to affect a large portion of the population and remains difficult to treat effectively in many cases, especially when gastrointestinal symptoms interfere with medication absorption. Atzumi, a dihydroergotamine mesylate nasal powder, recently received U.S. Food and Drug Administration approval and represents a promising alternative. It uses a unique powder-based delivery system aimed at improving absorption and consistency of effect. Data from both early pharmacokinetic testing and a long-term safety study suggest that it works quickly and is generally well tolerated. Patients experienced meaningful relief, often without needing backup medications, and side effects were reported infrequently.

## Introduction

Migraine is a globally prevalent, complex neurovascular disorder of the brain characterized by recurrent, severe headache attacks, usually of unilateral and pulsating quality, variably accompanied by nausea, vomiting, photophobia, and phonophobia^[1]^. The pathophysiology of migraine remains only partially understood, involving a multifactorial interplay of genetic, epigenetic, biochemical, and environmental factors^[2]^. Migraine affects approximately 10%–15% of the global population (around one billion people), making it the second leading cause of years lived with disability worldwide, particularly among the individuals aged 15–49 years^[3]^. In South Asia, including Pakistan, it remains underdiagnosed and undertreated, often compromising productivity and quality of life. While current acute treatments, such as oral triptans, dihydroergotamine (DHE), nonsteroidal anti-inflammatory drugs (NSAIDs), and ergot derivatives, offer relief for many patients, they are often limited by delayed onset, gastrointestinal side effects, and reduced efficacy during migraine-related gastroparesis^[4,5]^. NSAIDs are frequently associated with gastrointestinal and possibly cardiovascular side effects, ergots may induce arterial vasoconstriction, while triptans are contraindicated in cardiovascular, cerebrovascular, and peripheral vascular diseases^[5]^. In response to these limitations, the U.S. Food and Drug Administration (FDA) has recently approved a range of novel therapeutic options for both acute and preventive migraine management, including calcitonin gene-related peptide receptor antagonists, a selective serotonin receptor agonist, and noninvasive neuromodulation devices^[6]^. DHE exhibits distinct anti-migraine properties, including sustained efficacy even when administered long after the attack, effectiveness in the presence of central sensitization or cutaneous allodynia, low rates of migraine recurrence, and a reduced risk of medication overuse headache^[7]^.

Despite these advantages, the clinical utility of DHE has been constrained by its delivery methods, which traditionally require invasive administration (intravenous, intramuscular, or subcutaneous), and by the inconsistent pharmacokinetic profile and variable efficacy of intranasal formulations such as Migranal. The complex and time-consuming procedure of administration further contributes to its underutilization in acute migraine treatment^[7]^. Atzumi (formerly STS101), approved by the FDA on April 30, 2025, is a novel intranasal DHE powder formulation administered through a user-friendly, self-administered nasal device, designed for rapid absorption, offering a noninvasive, fast-acting alternative for patients needing effective acute migraine relief^[8]^. This article complies with the TITAN 2025 guidelines – representing the reporting and use of AI^[9]^.

## Mechanism of action

DHE acts primarily as an agonist at 5-HT1B, 5-HT1D, and 5-HT1F receptors, while also exhibiting affinity for 5-HT1A, 5-HT2A, and various adrenergic, cholinergic, and dopaminergic receptors, which may result in greater efficacy in patients inadequately responding to triptans^[10]^. Atzumi is the first and only intranasal DHE product to utilize the Simple MucoAdhesive Release Technology platform, which integrates a proprietary powder formulation and compact delivery device to enhance mucosal deposition, enabling rapid absorption through a steep concentration gradient and prolonged retention for sustained therapeutic effect^[8]^. Multiple studies have shown that a 5.2 mg dose of STS101 results in rapid and elevated plasma DHE levels within 20 minutes, with a favorable tolerability profile and higher systemic exposure than conventional liquid nasal DHE formulations^[11]^.

## Clinical development and FDA approval

Atzumi™ (DHE mesylate nasal powder) was produced through a comprehensive clinical program that spanned from kinetic assessments in the early stages to large safety trials. The Phase 1 trial was designed to evaluate the pharmacokinetics of Atzumi and demonstrated rapid absorption, attainment of high levels of DHE in plasma quickly, and low variation between doses^[11]^. These early findings helped confirm that the drug could relieve migraines at lightning speed. The core of the clinical development program was the ASCEND trial – the Phase 3 open-label long-term safety study of repeated as–needed dosing of Atzumi over 12 months in adults with migraine^[11]^. Taken together, the data from these studies provided support for the FDA approval of Atzumi as the first and only DHE nasal powder for the acute treatment of migraine with or without aura in adults^[8]^.

Clinical studies revealed that Atzumi can produce a prompt onset of therapeutic effect. A single, well-tolerated 5.2-mg DHE dose generated rapid and sustained plasma concentrations of DHE with low inter- and intrapatient variability: an important characteristic in the management of acute migraine, when rapid onset of action is important^[11]^. In the ASCEND study, 34.2% of patients were pain-free at 2 hours following treatment. Only 19.9% utilized their rescue medication, so most attacks were successfully managed without other additional treatment^[11]^.

In addition, the established safety profile of Atzumi over the long-term extends the clinical applicability. Atzumi was well tolerated during the 12 months of evaluation with no new safety findings identified following multiple applications^[11]^.

## Comparison to existing treatments

Atzumi brings several benefits over existing acute migraine treatments such as triptans and gepants, or earlier formulations of DHE nasal spray such as Migranal. In contrast to older DHE formulations, which exhibit non-predictable bioavailability and a delayed onset of action, Atzumi’s nasal powder is formulated to achieve more consistent and rapid systemic delivery^[11]^. The airborne powder technology showed faster absorption and less dose-to-dose variability. Atzumi had significantly better pharmacokinetic uniformity than Migranal, an attribute which is of particular benefit during migraines that require rapid treatment^[11]^.

### Safety profile

Atzumi exhibits a well-established safety profile, with voluminous long-term data from the ASCEND trial to support this. Symptom relief was very good at 2 hours of usage, adverse effects were rare, and it could be given with excellent tolerance even in repeated application^[11]^.

### Usability

Unlike older DHE sprays that required refrigeration and were difficult to prime, Atzumi is a powder-plus-device kit, more convenient to use, portable, and less cumbersome^[12]^. This ease of use may positively impact treatment adherence, particularly in patients with migraine-associated nausea or who require therapy away from home^[12]^.

Whilst formal cost-effectiveness analyses are pending, Atzumi’s clinical consistency and reduced use of rescue medication suggest possible economic benefits^[11]^.

## Limitations and considerations

Despite the favorable findings, long-term data remains limited, and further post-marketing surveillance is needed to fully establish safety, particularly regarding cardiovascular risk. Cardiovascular assessment is recommended prior to initiating Atzumi^[13]^.

## Conclusion

Atzumi combines a proprietary nasal powder formulation of DHE with an innovative nasal device to offer patients an effective, easy-to-use, portable treatment option for acute migraine attacks. Clinical studies demonstrated rapid absorption, high plasma DHE levels, sustained therapeutic effect, and favorable safety and tolerability^[11]^.

DHE has long been recommended as a first-line acute treatment option for migraine. Atzumi represents a significant advancement in delivery and patient convenience.

## Data Availability

Not applicable.
